# *Neisseria gonorrhoeae* Population Genomics: Use of the Gonococcal Core Genome to Improve Surveillance of Antimicrobial Resistance

**DOI:** 10.1093/infdis/jiaa002

**Published:** 2020-03-12

**Authors:** Odile B Harrison, Ana Cehovin, Jessica Skett, Keith A Jolley, Paola Massari, Caroline Attardo Genco, Christoph M Tang, Martin C J Maiden

**Affiliations:** 1 Department of Zoology, The Peter Medawar Building for Pathogen Research, South Parks Road, University of Oxford, Oxford, United Kingdom; 2 The Sir William Dunn School of Pathology, University of Oxford, South Parks Road, Oxford, United Kingdom; 3 Department of Immunology, Tufts University School of Medicine, Boston, Massachusetts, USA

**Keywords:** antimicrobial resistance, cgMLST, genome, gonorrhea

## Abstract

**Background:**

Gonorrhea, caused by the bacterium *Neisseria gonorrhoeae*, is a globally prevalent sexually transmitted infection. The dynamics of gonococcal population biology have been poorly defined due to a lack of resolution in strain typing methods.

**Methods:**

In this study, we assess how the core genome can be used to improve our understanding of gonococcal population structure compared with current typing schemes.

**Results:**

A total of 1668 loci were identified as core to the gonococcal genome. These were organized into a core genome multilocus sequence typing scheme (*N gonorrhoeae* cgMLST v1.0). A clustering algorithm using a threshold of 400 allelic differences between isolates resolved gonococci into discrete and stable core genome groups, some of which persisted for multiple decades. These groups were associated with antimicrobial genotypes and non-overlapping NG-STAR and NG-MAST sequence types. The MLST-STs were more widely distributed among core genome groups.

**Conclusions:**

Clustering with cgMLST identified globally distributed, persistent, gonococcal lineages improving understanding of the population biology of gonococci and revealing its population structure. These findings have implications for the emergence of antimicrobial resistance in gonococci and how this is associated with lineages, some of which are more predisposed to developing antimicrobial resistance than others.

## Teaser

Availability of gonococcal whole genome sequence data enhances our understanding of the population biology of this increasingly antimicrobial-resistant human pathogen. Here, genes core to the gonococcus are defined, which can be exploited to track emergence and transmission.

**(See the Editorial Commentary by Kahler on pages 1762–3 and the Major Article by Cehovin et al on pages 1826–36.)**

*Neisseria gonorrhoeae* is an obligate human bacterial pathogen. Colonization causes a localized inflammatory response, with untreated infections resulting in severe complications, ranging from disseminated infection to pelvic inflammatory disease. The impact on human health is exacerbated by the fact that gonococcal infection is asymptomatic in more than 60% of women and is a significant cause of infertility [1]. Prompt diagnosis and treatment are therefore essential; however, the gonococcus has developed resistance against all available classes of antimicrobials, such that options for the effective treatment of gonorrhea are becoming limited [2].

Understanding the evolution and persistence of antimicrobial resistance (AMR) in gonococci is critical to the successful control of gonorrhea. Antimicrobial resistance should be examined in the context of gonococcal population genomics, because this allows AMR emergence to be interpreted alongside the population structure and in association with genome content. This approach enables AMR to be recognized before transmission, potentially limiting expansion. Furthermore, this provides opportunities for predicting which gonococci may develop resistance and indicates how these variants can be controlled. However, the dynamics of gonococcal population biology is complex.

Gonococci have a fundamentally nonclonal population structure, a consequence of frequent intraspecies horizontal gene transfer (HGT) causing diversification and reassortment of variation over time [3]. Current molecular typing tools include the following: (1) multilocus sequence typing (MLST), which indexes the diversity found at 7 housekeeping gene fragments; (2) the *N gonorrhoeae* multiantigen sequence typing scheme, NG-MAST, where nucleotide sequence fragments of the outer membrane proteins PorB and TbpB are used to define NG-MAST sequence types; and (3) NG-STAR, a typing tool designed to track AMR [4–6].

Multilocus sequence typing relies on the characterization of fragments from housekeeping genes under stabilizing selection, and, before the advent of whole-genome sequencing, this was the method of choice for typing many bacterial species, including the related *Neisseria meningitidis* for which MLST was first developed [7]. Studies assessing the genetic diversity of gonococcal housekeeping genes have shown that, in addition to diversification arising from randomly distributed point mutations, these genes are frequently subject to HGT [8, 9]. As a result, some gonococci, although possessing the same 7 locus MLST-ST, will have different ancestry at the genome level. Nucleotide sequence fragments of both the porin gene, *porB*, and the transferrin binding protein B gene, *tbpB*, are used in the NG-MAST typing scheme [4]. Similarly, HGT among and within hypervariable *porB* and *tbpB* genes distorts relationships. The NG-STAR scheme indexes variability in nucleotide sequence fragments from 7 genes associated with AMR (*penA*, *mtrR*, *porB*, *ponA*, *gyrA*, *parC*, and the 23 rRNA gene) and can be used to determine chromosomally mediated AMR [10].

Availability of gonococcal whole genome sequence (WGS) data provides opportunities to understand the population biology of *N gonorrhoeae*. Here, gonococci were examined with a core genome MLST (cgMLST) scheme defined in this study, and core genome groups were used to identify related organisms. These analyses revealed the presence of loci core to the gonococcus, which remained stable over time within gonococcal groups. The gonococcal core genome scheme (*N gonorrhoeae* cgMLST v1.0) is available on the PubMLST *Neisseria* database (https://pubmlst.org/neisseria/) and can be can be used to track the emergence and persistence of gonococcal lineages, in combination with existing typing schemes MLST-ST, NG-MAST, and NG-STAR.

## METHODS

### Whole Genome Sequence Data and Assembly

Whole genome sequence data from 3750 *N gonorrhoeae* isolates, available on PubMLST (https://pubmlst.org/neisseria) [11], were included. This comprised published isolate collections and constituted a global dataset spanning 5 decades (1970 to 2018) [12–25].

Fastq reads were obtained from the European Nucleotide Archive (ENA) and assembled using the Velvet genome assembly program (v1.2.08) [26]. All odd-numbered kmer lengths were sampled for read lengths of 100, 125, and 150 base pairs, respectively, using the VelvetOptimiser software (v2.2.4) to automatically establish optimal Velvet assembly parameters. Resultant assemblies were deposited in the PubMLST *Neisseria* database, which uses the Bacterial Isolate Genome Sequence Database (BIGSdb) software [27].

### Definition of the Core Genome and Annotation

The core genome was derived using the Genome Comparator tool on PubMLST and the programs Prokka and Roary [28, 29]. Genome Comparator compares WGS data using either an annotated reference genome or a set of predefined loci [22]. Roary identifies bacterial pan-genomes using WGS data annotated with Prokka [28, 29]. The gene_presence_absence output file from Roary was used to identify loci present in >95% of the dataset, representing loci “core” to the gonococcus. This cutoff was chosen to account for the possibility that some loci may be stochastically absent in any given genome due misassembly, genome rearrangements, or mutations.

A blastn database of all loci and associated alleles defined in PubMLST was generated, after which core loci identified using Roary and Prokka were queried, allowing novel genes to be identified and defined in PubMLST. These were organized into the *N gonorrhoeae* cgMLST v1.0 scheme and the distribution verified using Genome Comparator. Genome Comparator allowed the most conserved and variable loci to be identified and *p-*distance values for each locus to be obtained. The R package ggplot2 was used to plot *p-*distance values per locus against locus length and visualized by function [30]. *P-*distance values indicate the proportion (p) of nucleotide sites at which 2 sequences compared are different. Genome Comparator can calculate *p-*distance values when multiple sequence alignments are generated. This option was chosen, and *p-*distance values were calculated locus-by-locus and between all isolates.

### Core Genome Sequence Type Clustering

Each isolate was annotated in as many of the loci included in the core genome as possible, resulting in core genome allele profiles, after which a core genome sequence type (cgST) was assigned. Such cgSTs are attributed daily with up to 50 loci within the core genome scheme allowed to be “missing” due to incomplete or absent sequence data. Such loci are designated with an “N” representing any allele. Single-linkage clustering was applied to each cgST, grouping these using increasing allelic difference thresholds. For example, using an allelic threshold of 5 locus differences, isolates with cgST profiles differing with at least 1 other profile at 5 loci or fewer clustered into a core genome group. Correspondingly, isolates belonging to this core genome group would share identical alleles across the remaining 1662 loci of the core genome with another isolate in this group. Increasing thresholds of allele differences were applied ranging from 5 or fewer allelic differences to 10, 25, 50, 100, 200, 300, 400, and 500.

### Multilocus Sequence Typing, NG-MAST, and NG-STAR

The MLST-STs were assigned using the scheme devised for *N meningitidis*. The NG-MAST scheme was implemented in PubMLST. The POR and TBPB loci defined in NG-MAST.net were defined as NG-MAST_porB and NG-MAST_tbpB. Corresponding NG-MAST STs were implemented. The NG-STAR scheme was also established in PubMLST and mirrors the scheme hosted at https://ngstar.canada.ca [6]. To identify loci with NG-STAR, the nomenclature used in PubMLST was as follows: “mtrR, NG_porB, NG_ponA, NG_gyrA, NG_parC, and NG_23S. The *penA* sequence used in NG-STAR is identical to NEIS1753.

A total of 87 NG-MAST *tbpB* alleles, defined on www.ng-mast.net [4], were found to be either identical shorter nucleotide sequence fragments nested within previously defined alleles, truncated, reverse-complemented, or less than 70% identical to previously defined *tbpB* alleles and were retired. A total of 149 *porB* NG-MAST alleles were also retired because these were found to be less than 70% identical to defined *porB* alleles or were identical subsequences of defined alleles. This resulted in the withdrawal of 432 NG-MAST STs. A total of 623 new NG-MAST STs were defined.

### Phylogenetic Analyses

GrapeTree clusters isolates based on their allelic profiles, using a minimum spanning algorithm. Isolates were compared using the *N gonorrhoeae* cgMLST scheme and the resulting trees annotated by core genome group at thresholds 300, 400, and 500. Previous studies have used hierarchical Bayesian analyses (BAPS) to identify gonococcal lineages [19, 25, 31]. A dataset of 419 gonococci published previously were analyzed using rhierBAPS [19, 32]. To do this, a concatenated core genome alignment was obtained using Genome Comparator. This was used as the input sequence alignment in rhierBAPS. Clustering was performed with 2 levels in the hierarchy using k = 40 as the prior upper bound for the number of clusters. A maximum likelihood tree was generated using PhyML [33], and both core genome groups identified from cgMLST analyses and BAPS-derived lineages were annotated onto the maximum likelihood tree using Evolview [34].

## RESULTS

### Dataset and Genome Features

The dataset included WGS dating from the 1970s to 2018 and from Africa (157) [15, 23], Asia (278) [18, 20], Europe (2234) [14, 16, 19], North America (843) [12, 13, 21], Oceania (128) [25], and South America (100) [17]. Ten isolates were from unknown countries. Reference genomes from FA1090, NCCP11945, and TCDC-NG08107 and complete genome data from 12 isolates were included [35]. The remaining WGS data were Illumina-derived, high-quality, draft sequence assemblies with the number of contigs ranging from 37 to 188. Accession numbers and genome assembly statistics can be found in Supplementary Table 1 (https://figshare.com/s/e1486de145f709d7434d).

A total of 32 isolates did not have an MLST-ST, due to incomplete profiles. Of the remaining isolates, 292 MLST-STs were identified. The most frequent ST was ST-1901 (915 of 3718, 25%) (Supplementary Table 1). NG-MAST 1407 was the most prevalent ST (326 of 3736, 9%) with a total of 383 isolates possessing NG-MAST STs occurring once only. A total of 556 NG-STAR STs were found, with NG-STAR ST-90 the most prevalent (365 of 3217, 11%). A total of 83 NG-STAR STs were present in pairs of isolates with a further 313 STs occurring once. NG-STAR STs could not be defined in 357 isolates due to incomplete or absent loci in either of the 7 loci in the scheme.

MLST and NG-MAST STs defined in PubMLST were compared with a published dataset [19]. Agreement between MLST and NG-MAST STs was apparent in the majority of cases. Discrepancies were due either to (1) an isolate lacking an ST in PubMLST due to the presence of incomplete alleles resulting from these located at the end of a contig or (2) isolates assigned MLST-ST 1024 in the published dataset of which there were 65 (Supplementary Table 2). These dated from 1989 to 2012, originated from 26 countries, and were all ST-1024; however, different MLST-STs were found here (Supplementary Table 2) (https://figshare.com/s/4be06cdd071a2e9eb59e). Only 1 other isolate record was found in PubMLST with this ST. This belonged to an isolate from the Czech Republic dating from 2001; however, accompanying WGS data were not available. Phylogenetic analysis of the distribution of the ST-1024 isolates revealed that these were not associated with specific core genome groups (Supplementary Figure 1). The same isolate records in Pathogenwatch (https://pathogen.watch) possessed MLST-STs identical to those listed in PubMLST.

### Core Genome Characteristics

**Figure 1.**
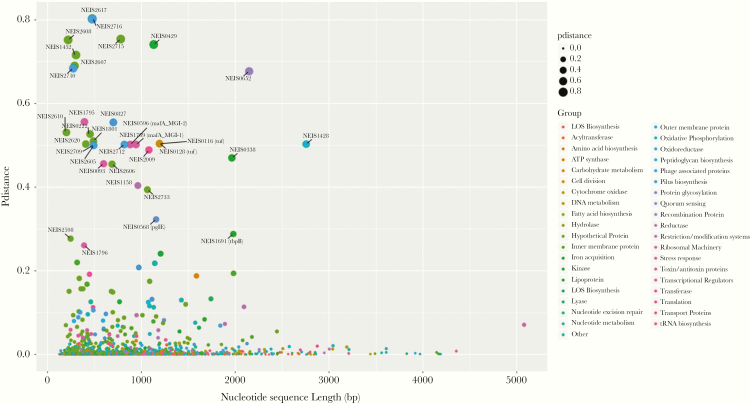
Core loci diversity. Diversity of core genome loci plotted against nucleotide sequence length (base pairs [bp]). Loci are color coded by functional group; for example, olive green circles represent hypothetical protein genes. Circle size is proportional to the amount of diversity. It can be seen that much of the core genome is highly conserved including loci >4000 bp in length.

A total of 1668 loci were defined as core to the gonococcal genome in this dataset. Allelic designations were defined for approximately 98% of the core genome with 98% of loci annotated in all isolates (Supplementary Table 1). The most conserved loci were NEIS0415 (ribosome biogenesis GTP-binding protein YsxC, NGO0100) and NEIS2686 (hypothetical protein, NGO1147). A further 1529 loci had *p-*distance values ≤0.02, followed by 79 loci possessing *p-*distance values ranging from 0.03 to 0.1. This included the following: 31 hypothetical proteins; the iron acquisition proteins TbpA (NEIS1690) and FetA (NEIS1963); proteins implicated in pilin biosynthesis (NEIS0411 [*pilN*], NEIS0487, NEIS0828, NEIS0830, NEIS0831, and NEIS1995); protein glycosylation NEIS0399 (*pglB*); restriction modification systems (NEIS0328 [*dpnIIB*], NEIS2362, NEIS2535, and NEIS2595); and toxin-antitoxin subunits (NEIS0591, NEIS0593, and NEIS2101). Higher *p-*distance values (*p-*distance = 0.12 to 0.3) were observed in a further 32 loci with 26 loci identified with *p-*distance values ranging from 0.4 to 0.8 (Figure 1). These were associated with pilin biosynthesis (NEIS0827 *p-*distance = 0.555), cell division (NEIS0116 and NEIS0128 [*tuf*] *p-*distance = 0.504), and iron acquisition (NEIS0338 *p-*distance = 0.47) and included 10 hypothetical proteins (NEIS0222, NEIS1452, NEIS1801, NEIS2605, NEIS2606, NEIS2607, NEIS2608, NEIS2610, NEIS2620, and NEIS2715).

### *Neisseria gonorrhoeae* Core Genome Clusters 

Core genome groups are designated with “Ng_cgc_”, indicative for *N gonorrhoeae* core genome group cluster, followed by the locus difference threshold used, eg, Ng_cgc_5 for a threshold of 5 or fewer loci. A total of 2909 Ng_cgc_5 core genome groups were identified. Of these, 2265 comprised single isolates, with the remaining groups including paired groups of isolates and a single group consisting of 47 isolates. These dated from 2004 to 2007 and were from the United Kingdom. The majority of these isolates formed part of a study examining 2 gonorrhea outbreaks in the United Kingdom [24], the exception being 2 isolates that formed part of a separate study [14].

**Figure 2.**
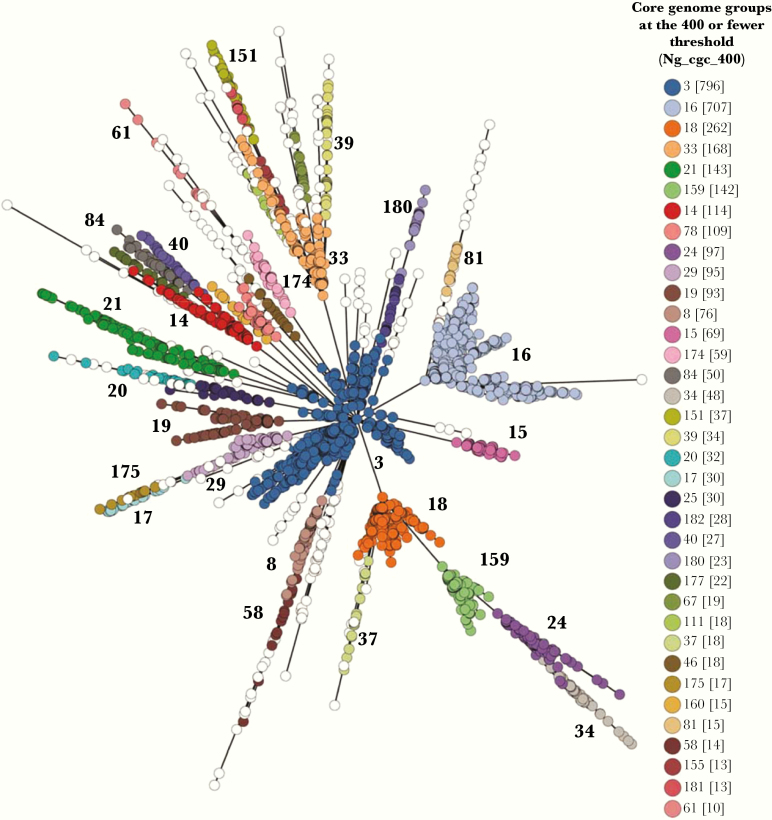
Minimum spanning tree comparing core genome allelic profiles in association with Ng_cgc_400 core genome groups. Whole genome sequence data were compared using GrapeTree resulting in isolates with similar allelic profiles forming clusters. These were then annotated by core genome group following the 400 or fewer locus threshold. Core genome groups with less than 10 isolates are represented with white circles. Numbers in brackets refer to the number of isolates belonging to that core genome group. Ng_cgc_400 core genome groups are also displayed next to each cluster.

A total of 2406 Ng_cgc_25 core genomes were observed, with 1769 groups composed of unique isolates. At the 50-locus differences or fewer threshold, 1042 core genome groups were observed, decreasing to 675 core genome groups at the 100-locus threshold. Greater resolution was obtained using a 200 or fewer locus threshold, reducing to 261 core genome groups at the 300-locus threshold, with 196 groups at the 400 threshold. Using a core genome threshold of 400 or fewer locus differences, we identified distinct core genome groups indicative of related groups of gonococci (Figure 2). These were also found to persist over time (Supplementary Figure 2).

The most prevalent core genome group at this threshold, Ng_cgc400_3, was globally distributed. This was also the case for core genome groups 16, 18, and 159. Ng_cgc400_ 34 was identified in gonococci originating from Kenya and India only, in which AMR was found to be plasmid-mediated [15]. Distinct core genome groups were also apparent for Australia, Bhutan, China, Estonia, Pakistan, and the Philippines.

To identify the optimal core genome locus threshold to be used, core genome groups resulting from 300-, 400-, and 500-locus thresholds were mapped onto minimum spanning trees (Supplementary Figure 3). A number of additional core genome groups were observed using the 300 threshold (Supplementary Figure 3A), whereas at the 500 threshold, larger core genome groups were found, resulting in less resolution (Supplementary Figure 3C). Based on these results, a threshold of 400 or fewer locus differences was chosen for further analysis (Supplementary Figure 3B).

### Associations Between Ng_cgc_400 Core Genome Groups and Current Typing Schemes

**Figure 3.**
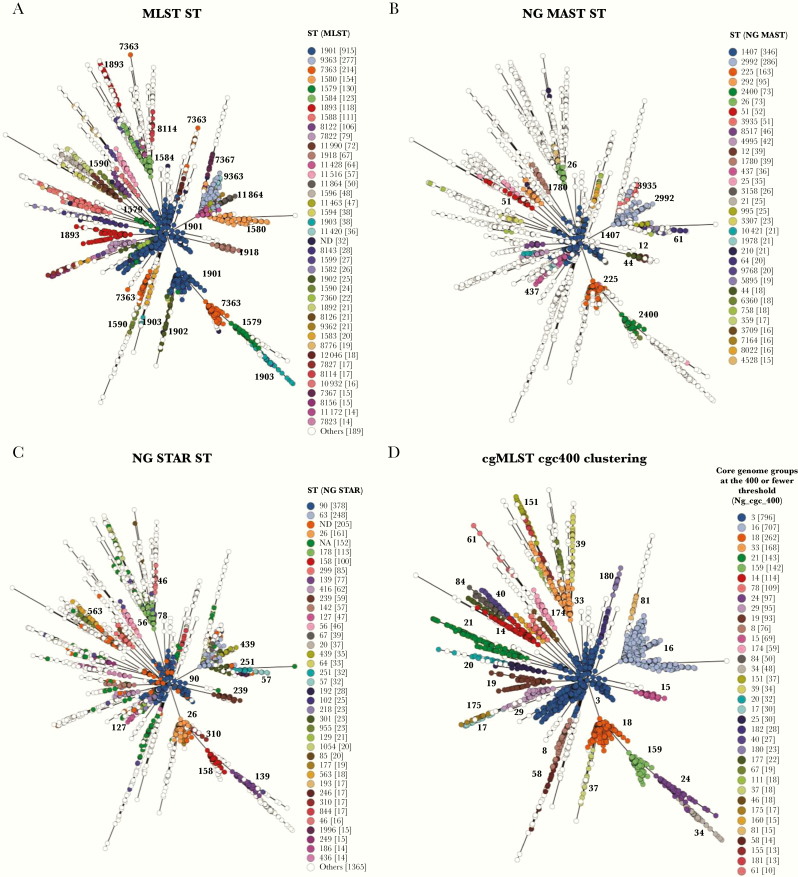
Minimum spanning tree comparing core genome allelic profiles in association with *Neisseria gonorrhoeae* typing schemes. Whole-genome sequence data were compared using the core genome and annotated by multilocus sequence type (MLST)-ST (A), NG-MAST ST (B), NG-STAR ST (C), and Ng_cgc400 core genome groups (D). Legends only depict groups containing 10 or more isolates. NA refers to the unavailability of an ST due to gene absence or inability to assign an allele, whereas ND refers to an ST that has not been determined due to an incomplete ST resulting from incomplete allelic profile.

Core genome group Ng_cgc400_3 included all gonococci belonging to NG-STAR ST-90 (365 of 796, 46%) and NG-STAR ST-127 (31 of 796, 4%) (Figure 3). NG-STAR ST-127 was concomitant with NG-MAST STs 337 (Japan n = 4) and 2018 (Brazil n = 4). Ng_cgc400_16 included gonococci belonging to NG-STAR ST-63 (248 of 707, 35%), whereas Ng_cgc400_24 included NG-STAR ST-139 (76 of 97, 78%). Ng_cgc400_84 included gonococci belonging to NG-STAR ST-955 (United Kingdom = 23) and 307 (United Kingdom = 6; Poland = 1) (Figure 3B and C). MLST-STs were more dispersed. For example, MLST-ST 1901 gonococci belonged to Ng_cgc400_3 and _18, MLST-ST 7363 belonged to Ng_cgc400_8, _108, _132, _150, _159, _180, and _187, and MLST-ST 1579 belonged to Ng_cgc400_3, _18, _24, and _81 (Figure 3A).

### Phylogenetic Analyses

Minimum spanning trees were generated using GrapeTree implemented in PubMLST [36]. This tool clustered isolates according to allelic profiles in the core genome. Isolates were then annotated by Ng_cgc300, Ng_cgc400, or Ng_cgc500 core genome groups (Supplementary Figure 3). The published dataset [19] comprising 419 isolates was analyzed and annotated with both Ng_cgc400-derived core genome groups and rhierBAPS-derived groups (Supplementary Figure 4). Consistent with previous results, 9 BAPS derived clades were found located in multiple regions of the tree. This was in contrast to core genome groups that formed distinct clusters.

## DISCUSSION

The gonococcus is capable of developing AMR using chromosomally and/or plasmid-mediated mechanisms [37]. Therefore, a combination of approaches is needed to (1) delay the emergence and increase of AMR, (2) rapidly detect AMR variants, and (3) prevent transmission and expansion. To this end, a number of resources have been developed, allowing AMR to be detected from WGS. This includes tools such as ABRicate [38], PAARSNP (implemented in the online tool Pathogenwatch) [39], and Gen2Epi; the latter is an online pipeline allowing WGS to be assembled and linked to AMR profiles [40]. However, our understanding of the gonococcal population biology remains incomplete and is obscured by the presence of a complex structure with frequent HGT. In this study, we describe how the core genome can be used to improve resolution of the gonococcal population structure.

Extensive HGT in housekeeping genes, including those used in MLST, precludes the use of small numbers of loci to robustly examine gonococcal population structure (Figure 3A) [8]. This is in contrast to the related *N meningitidis*, for which the population structure of isolates associated with invasive disease can be readily distinguished using MLST-STs organized into clonal complexes [41]. In the meningococcus, the higher level of linkage disequilibrium of MLST loci results in the nonrandom association of allelic profiles producing discrete meningococcal lineages, which persist over time and remain fixed with the core genome [42, 43]. Surveillance of invasive meningococcal disease outbreaks relies on defined strain designations including MLST-STs, hyperinvasive clonal complexes, and capsular serogroups, allowing preventative measures including vaccines to be deployed [44]. In contrast, due to HGT, gonococcal MLST-STs are distributed throughout the population and are associated with several core genome groups (Figure 3A). This indicates that combinations of MLST alleles in gonococci are unlikely to be associated with transmission fitness or positive selection.

It has long been known that identifiable gonococcal genetic variants can persist over time with, for example, the arginine, hypoxanthine, uracil-requiring (AHU-) gonococcal auxotrophs [45], indicating that alternative phenotypic selection pressures, involving metabolic processes and/or AMR, are exerted. Core genome clustering identified discrete groups of gonococci, some of which have persisted over time, consistent with our analyses (Figure 2 and Supplementary Figure 2). Non-overlapping NG-STAR or NG-MAST profiles were associated with core genome groups. For example, NG-STAR 90, which is known to consist of gonococci with reduced susceptibility to cephalosporins and ciprofloxacin, was found only with gonococci belonging to Ng_cgc400_3 (Figure 3) [6]. NG-MAST STs were also associated with core genome groups (Figure 3B). The genetic markers used in NG-STAR and NG-MAST encode particular phenotypes with NG-STAR representing AMR and NG-MAST outer membrane proteins subject to immune selection [6, 46, 47]. These data indicate that combinations of alleles across the core genome are associated with transmission fitness and are likely subject to selection. This will lead to persistence of core genome groups in association with discrete non-overlapping NG-STAR and NG-MAST allelic profiles. Therefore, identifying to which core genome group gonococci belonged will improve detection of AMR variants.

Previous studies assessing the population structure of gonococci have used hierarchical BAPS [31] that, using SNP-based analyses, generates clusters after accounting for HGT [48]. In this study, rhierBAPS was used to identify clades, which were compared with gonococcal core genome groups (Supplementary Figure 4). Consistent with previous analyses, 9 BAPS-derived clades were found [19]. Congruence of the larger clades with core genome groups was observed; however, greater resolution was apparent using core genome clustering with the presence of distinct groups of gonococci (Supplementary Figure 4). The use of core genome groups, as defined on PubMLST, has several advantages compared with single-nucleotide polymorphism-based analyses. The clustering algorithm is run daily, such that any new data deposited in the database, or gonococci for which cgSTs have not yet been defined, will be automatically assigned to a core genome group in combination with NG-STAR, NG-MAST, and MLST-STs. This will allow datasets to be continually compared, without the need for referring back to raw data or the use of a reference genome. This also allows the diversity across the core genome to be assessed in increasingly larger datasets. Indeed, analyses undertaken here revealed that a large proportion of the core genome remained highly conserved (Figure 1).

Implementation of core genome clustering algorithms using low or high locus difference thresholds has the potential to allow transmission networks to be determined from WGS. Previous estimates of gonococcal evolution have estimated that approximately 3.6 nucleotide polymorphisms occur per year per genome, allowing the relationships among gonococci to be determined [14]. Such analyses have identified highly related gonococci differing in only 1 polymorphic site [49]. Further work is necessary to investigate whether core genome clustering thresholds can be used to identify transmission networks. As increasing amounts of WGS become available, it is possible for some core genome groups to merge. For this reason, fixed, central core genotypes will need to be defined to provide a stable nomenclature with which variants within the population can be reliably tracked. Such core genotypes will need to be identified in as wide a dataset as possible to ensure that the diversity of the gonococcal population is represented.

## CONCLUSIONS

The related meningococcus has been described as an “epidemic clone” with lineages associated with invasive disease persisting for decades or longer against a background of diverse meningococci found in asymptomatic carriage [50]. In contrast, the gonococcus can be thought of as a “sexual clone.” In this case, a single organism founded the gonococcal population; however, frequent within-species HGT has led to genetic reassortment and diversity. Nevertheless, persistent groups of gonococci with distinct core genomes can be found. Identification and comparison of the core genome using cgMLST will allow the emergence and persistence of global gonococcal lineages to be monitored and will improve our understanding of the population structure of this human pathogen in a reliable and reproducible manner.

## Supplementary Data

Supplementary materials are available at *The Journal of Infectious Diseases* online. Consisting of data provided by the authors to benefit the reader, the posted materials are not copyedited and are the sole responsibility of the authors, so questions or comments should be addressed to the corresponding author.

**Supplementary Figure 1.** The dataset published by Sánchez-Busó et al [19] was compared using the core genome. A depicts isolates labeled by MLST ST. Only STs in which 3 or more isolates belonging to that ST have been labeled. B depicts isolates found to be ST-1024 in the Sánchez-Busó et al [19] paper. It can be seen that these isolates are randomly distributed throughout the tree. Numbers in brackets refer to the number of isolates belonging to that ST.

**Supplementary Figure 2.** Persistence of core genome groups over time. Using Tableau, the number of isolates belonging to each core genome group were plotted against the year in which they had been isolated. From this it was apparent that a number of core genome groups have persisted for several decades.

**Supplementary Figure 3.** Comparison of core genome groups obtained using thresholds 300, 400, and 500. Whole genome sequence data were compared using the core genome and annotated by core genome groups obtained using the 300 or fewer locus difference threshold (A), the 400 or fewer locus difference threshold (B), or the 500 or fewer locus difference threshold (C). From this it was observed that a number of additional core genome groups were found using the 300 threshold, eg, groups 26 and 69 (A), whereas at the 500 threshold larger core genome groups were found resulting in less resolution, eg, group 3 (C). Based on these results, a threshold of 400 or fewer locus differences was determined to be the optimum threshold for investigating gonococcal populations (B).

**Supplementary Figure 4.** Maximum likelihood tree depicting rhierBAPS-derived clades and cgc400 core genome groups. The dataset of 419 gonococci originating from the Sánchez-Busó et al [19] paper were analyzed using rhierBAPS [32]. Both Ng_cgc_400 core genome groups and BAPS-derived lineages were annotated onto a maximum likelihood tree generated from the concatenated core genome alignment. Consistent with previous results, 9 rhierBAPS-derived clades were found (inner ring), and these were located in multiple regions of the tree. This was not the case with core genome groups, which remained distinct (outer ring). Ng_cgc_400 core genome groups are indicated next to each cluster where these contained 3 or more isolates.

**Supplementary Table 1.** Isolate dataset. Table depicting the isolate dataset used including accession numbers, typing schemes, references, and whole genome assembly statistics. This table can be downloaded by following this link: https://figshare.com/s/e1486de145f709d7434d.

**Supplementary Table 2.** Comparison with MLST ST-1024 isolates. Table depicting isolates obtained from the Sánchez-Busó et al [19] paper that were found to be MLST ST-1024. This table can be downloaded by following this link: https://figshare.com/s/4be06cdd071a2e9eb59e.

jiaa002_suppl_Supplementary_Figures

## Notes

***Financial support.*** O. B. H., J. S., and M. C. J. M. were funded by Wellcome Trust Grant 214374/Z/18/Z; K. A. J. was funded by Wellcome Trust Grants 104992/Z/14/Z and 218205/Z/19/Z; C. M. T. and A. C. were funded by the Oxford Martin School and Wellcome Trust (Grant Numbers 102908/Z/13/Z and 214374/Z/18/Z); C. A. G. was funded by National Institutes of Health (NIH) Grants R01AI116969 and R01 AI130946; P. M. was funded by NIH Grant R21AI131004.

***Potential conflicts of interest.*** All authors: No reported conflicts of interest. All authors have submitted the ICMJE Form for Disclosure of Potential Conflicts of Interest.
