# Supplementary material for: Neisseria gonorrhoeae Population Genomics: Use of the Gonococcal Core Genome to Improve Surveillance of Antimicrobial Resistance
Source: J Infect Dis. 2020 Mar 12;222(11):1816–25. doi: 10.1093/infdis/jiaa002 (PMC7653085; doi:10.1093/infdis/jiaa002)
Supplement: jiaa002_suppl_Supplementary_Figures [file jiaa002_suppl_supplementary_figures.pptx]

## Slide 1
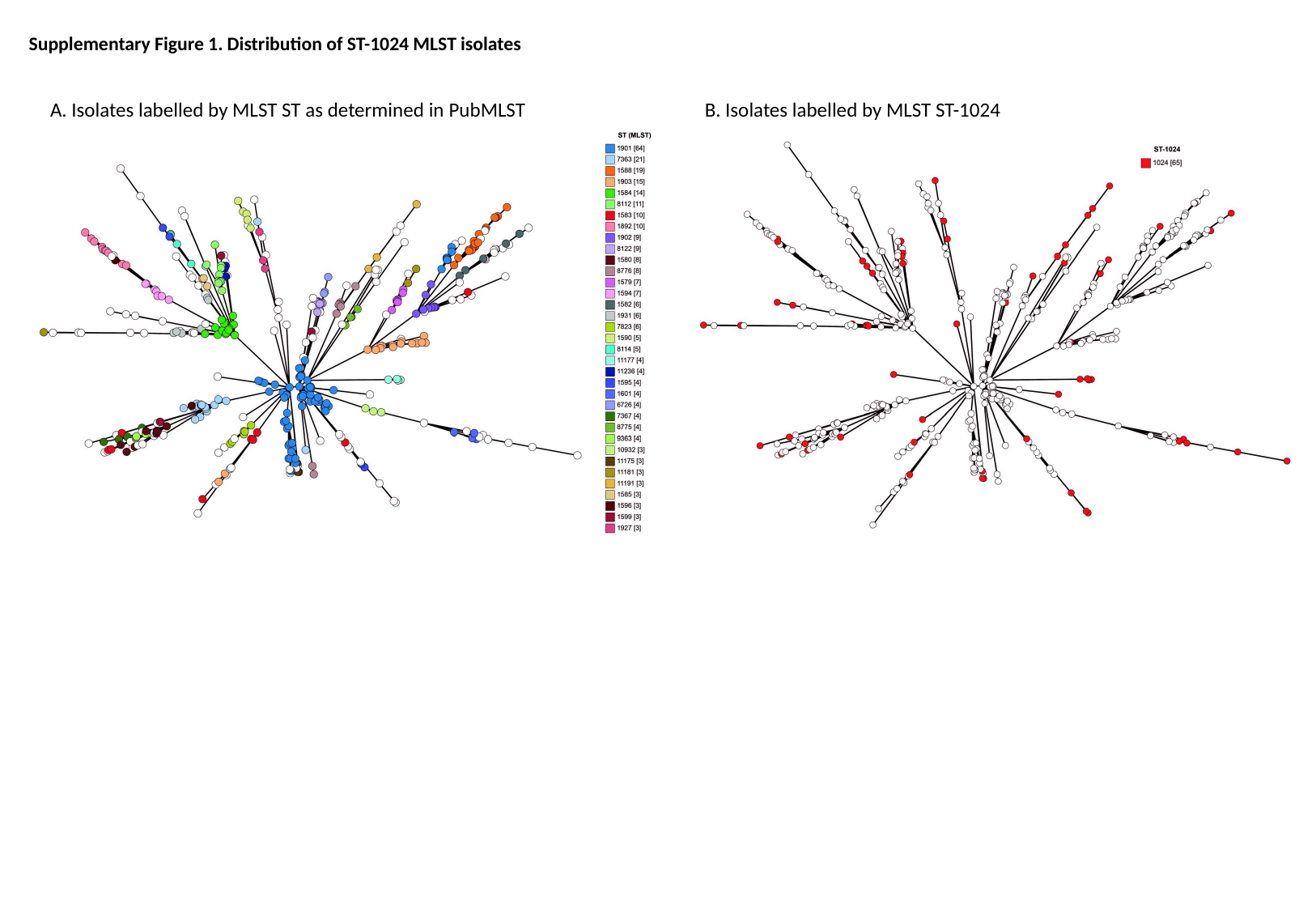

Supplementary Figure 1. Distribution of ST-1024 MLST isolates
B. Isolates labelled by MLST ST-1024
A. Isolates labelled by MLST ST as determined in PubMLST

## Slide 2
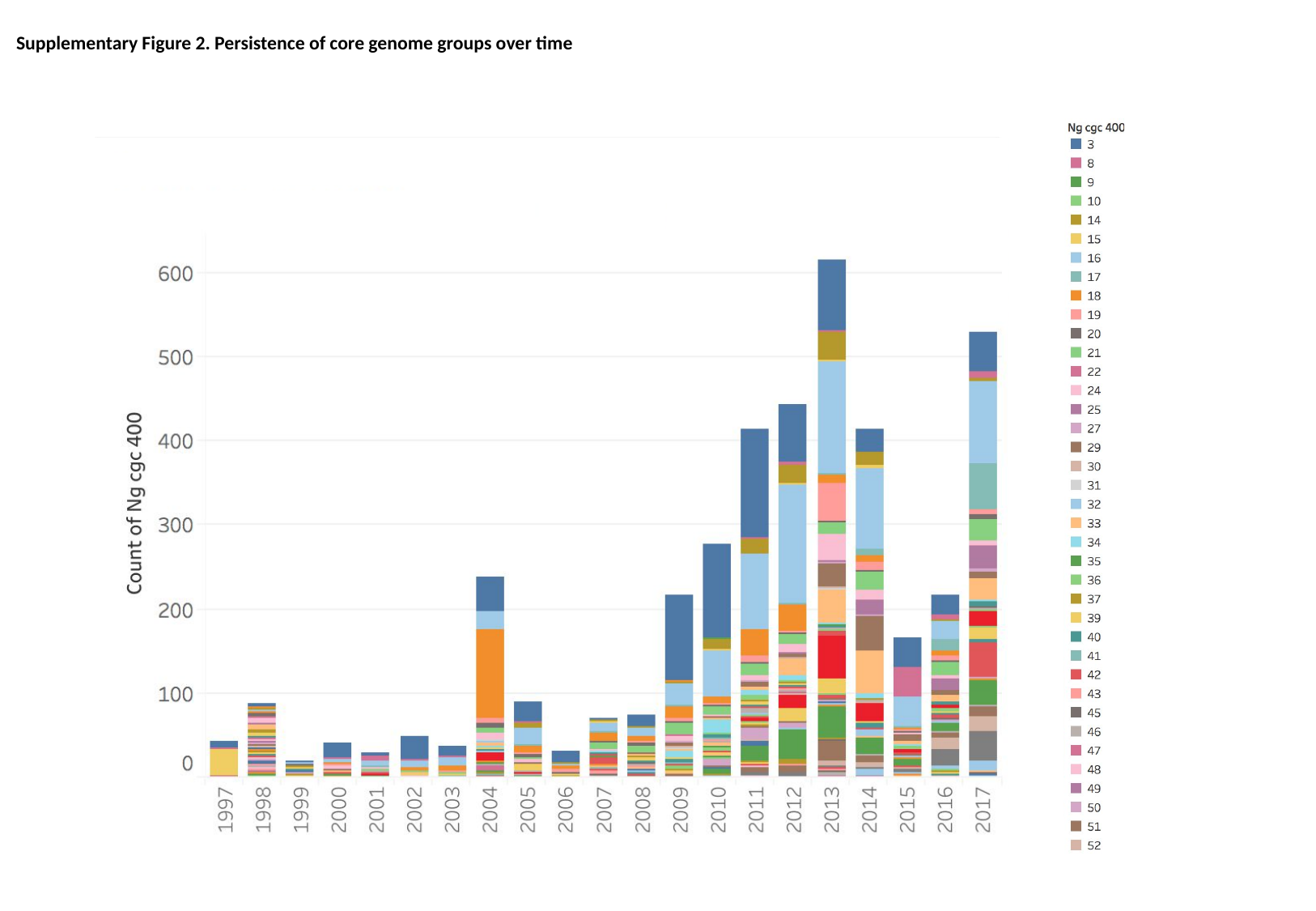

Supplementary Figure 2. Persistence of core genome groups over time

## Slide 3
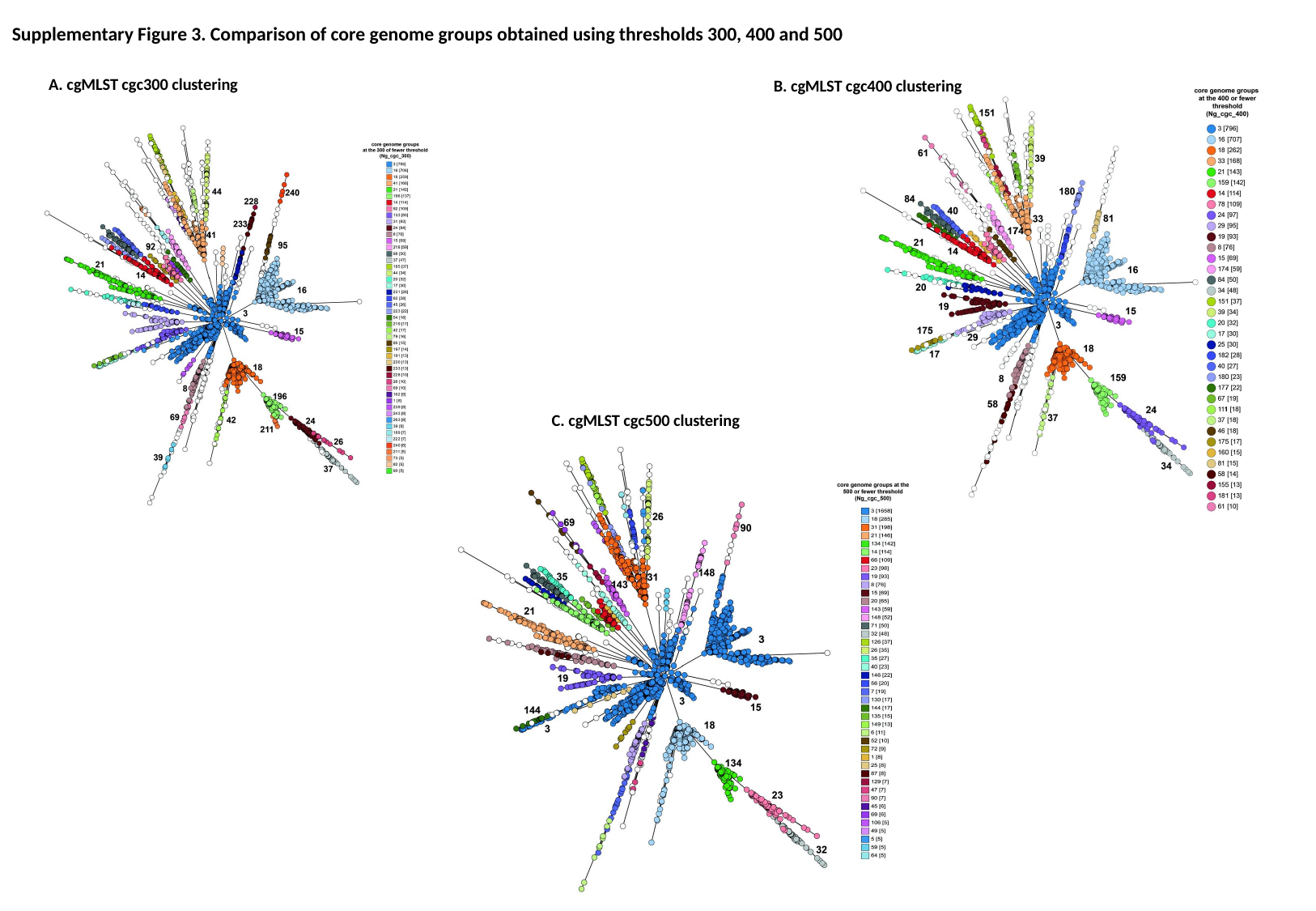

Supplementary Figure 3. Comparison of core genome groups obtained using thresholds 300, 400 and 500
A. cgMLST cgc300 clustering
B. cgMLST cgc400 clustering
C. cgMLST cgc500 clustering

## Slide 4
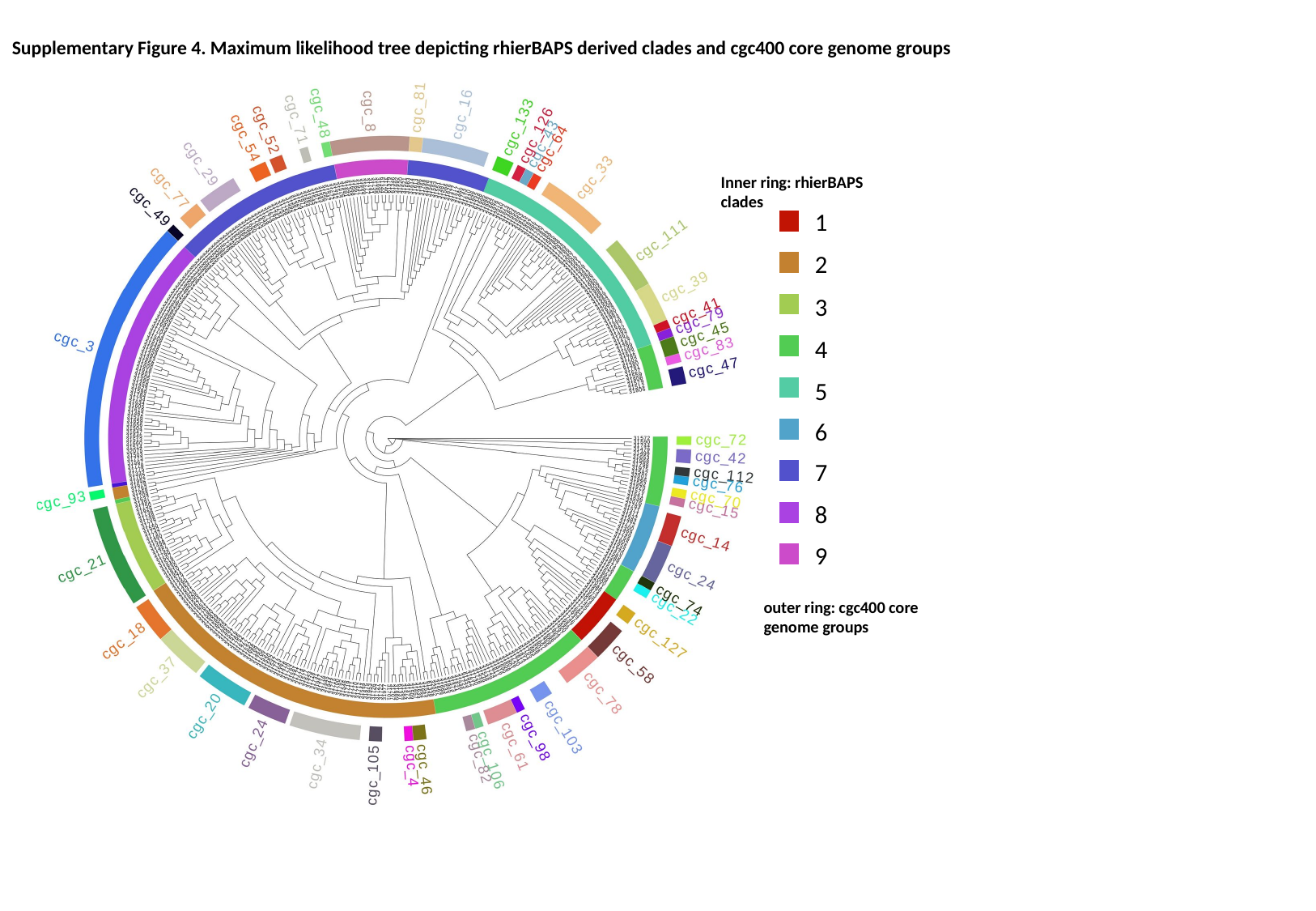

Supplementary Figure 4. Maximum likelihood tree depicting rhierBAPS derived clades and cgc400 core genome groups
Inner ring: rhierBAPS clades
1
2
3
4
5
6
7
8
9
outer ring: cgc400 core genome groups
